# Effect of ginsenoside compound K on alleviating colitis via modulating gut microbiota

**DOI:** 10.1186/s13020-022-00701-9

**Published:** 2022-12-28

**Authors:** Li Wang, Li Shao, Man-Yun Chen, Lin Wang, Wei Zhang, Feng-Bo Tan, Wei-Hua Huang

**Affiliations:** 1 Department of Clinical Pharmacology, Xiangya Hospital, Central South University, Changsha, 410008 China; 2 Institute of Clinical Pharmacology, Hunan Key Laboratory of Pharmacogenetics, Central South University, Changsha, 410078 China; 3 National Clinical Research Center for Geriatric Disorders, Xiangya Hospital, Central South University, Changsha, 410008 China; 4 Department of Pharmacognosy, School of Pharmacy, Hunan University of Chinese Medicine, Changsha, 410128 China; 5 Department of General Surgery, Xiangya Hospital, Central South University, Changsha, 410008 China

**Keywords:** Ginsenoside compound K, Colitis, FMT, Gut microbiota

## Abstract

**Background:**

Ginsenoside compound K (GC-K) potentially alleviates ulcerative colitis involved in gut microbiota, which is significantly associated with the occurrence and development of colitis. However, the effect and mechanism of GC-K on anti-colitis in relation to gut microbiota are not clear. This study focused on the prevention and mechanism of GC-K on Dextran sulfate sodium (DSS)-induced colitis of mice pertinent to gut microbiota.

**Methods:**

DSS was used to establish a chronic colitis mouse model. Body weight analysis, colon length measurement, HE staining, and inflammatory factors levels were processed in animal experiments. Flow cytometry was employed to analyze Th17/Treg cells in the mouse spleen and blood. 16S rRNA sequencing was utilized to analyze gut microbiota. Fecal microbiota transplantation (FMT) experiment was employed to verify the anti-colitis efficacy of GC-K by reshaping gut microbiota.

**Results:**

GC-K significantly relieved colitis-related symptoms due to decreased disease activity index (DAI) scores, spleen weight, and increased colon length. Additionally, the tight junction proteins were increased, and the pro-inflammatory cytokines, such as TNF-α, IL-6, IL-1β and IL-17, were decreased after GC-K treatment. Furthermore, *Bacteroides* spp. significantly increased after modeling. Moreover, FMT experiments confirmed that GC-K-driven gut microbiota greatly relieved DSS-induced colitis.

**Conclusion:**

GC-K alleviated colitis via the modulation of gut microbiota.

**Graphical Abstract:**

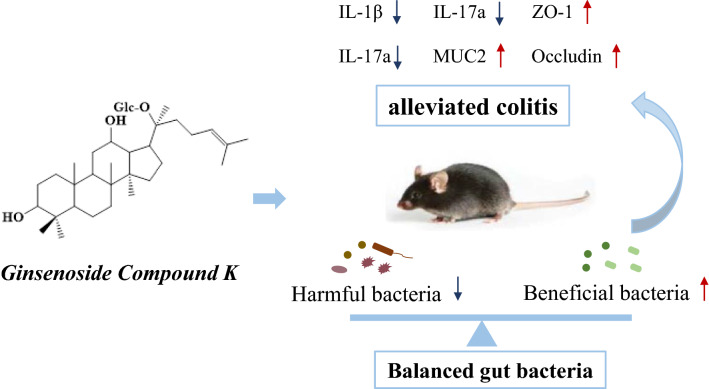

**Supplementary Information:**

The online version contains supplementary material available at 10.1186/s13020-022-00701-9.

## Highlights

- *Ginsenoside Compound K* (GC-K) alleviated colitis in DSS-induced Mice.
- GC-K modulated gut microbiota in DSS-induced mice.
- GC-K relieved colitis via regulating gut microbiota.
- GC-K-driven gut microbial effectively ameliorated colitis.

**Supplementary Information:**

The online version contains supplementary material available at 10.1186/s13020-022-00701-9.

## Introduction

Ulcerative colitis (UC), a chronic nonspecific intestinal inflammatory disease, clinically manifests as abdominal pain, bloody diarrhea, and even degrees of systemic symptoms, becoming a global disease in gastroenterology [1, 2]. Colitis triggers inflammatory cell infiltration followed by cytokine release syndrome and systemic inflammation. The pathological mechanism of colitis is not well-known, so colitis is symptomatically treated in the clinic along with adverse effects and easy recurrence. Therefore, it is essential to develop safe and effective drugs for colitis.

Although the underlying pathogenesis of UC is incompletely understood, numerous studies have demonstrated that gut microbiota plays a vital role in the pathogenesis of UC. Intestinal inflammation disappears in a spontaneous model of colitis in germ-free IL-10^(−/−)^ mice [3, 4]. Meanwhile, the gut microbiota of UC patients maintains dysbiosis by comparing with healthy humans. The α-diversity of gut microbiota and some beneficial gut microbes, such as *Faecalibacterium prausnitzii* and *Akkermansia muciniphila*, were reduced in UC patients. *F. prausnitzii* can destroy the activation of the NF-κB pathway and block the production of IL-8 by producing butyrate, while *A. muciniphila* ameliorates colitis by regulating immune responses and gut barrier repair [5–7]. On the contrary, the abundance of pathogenic bacteria was increased, e.g., *Bacteroides vulgatus*, which exacerbates UC through secreting proteases [8]. Due to gut microbiota dysbiosis as a pathogenic factor that leads to an impaired intestinal barrier and promotes the onset of UC, targeting the gut microbiota could be a practical therapeutic approach for preventing and curing colitis.

Ginsenoside compound K (GC-K) is one of the primary metabolites of ginseng saponins bio-converted by gut microbiota in vivo [9–11], which possesses various pharmacological activities, such as anti-diabetic, anti-hepatic lipid accumulation, anti-inflammation, and antitumor effects. In addition, GC-K ameliorates colitis and inhibits inflammatory responses by suppressing NF-kB activation [12]. However, the absolute oral bioavailability of GC-K is only about 35% [13]. GC-K is catalyzed by *β*-glycosidase only secreted from the gut microbiota to generate its metabolite protopanaxadiol in vivo, which implies that GC-K interplays with gut microbiota after oral administration [14]. Furthermore, our previous study found that GC-K could suppress the tumor growth of AOM/DSS-induced colitis-associated colorectal cancer through the modulation of gut microbiota, partially by the up-regulation of *A. muciniphila* [9]. Accordingly, we hypothesized that GC-K could soothe colitis by regulating gut microbiota.

In this study, the effect of GC-K on gut microbiota was explored in DSS-induced colitis to verify whether gut microbiota mediated the progression. We observed that GC-K ameliorated experimental colitis and regulated gut microbial such as *Bacteroides *spp., which were enriched in the colitis group. In addition, co-incubation experiments further demonstrated the regulatory effect of GC-K on *Bacteroides *spp. Spearman correlation analysis showed that the progression of the UC phenotype was related to gut microbiota. To verify the beneficial effects of treatment-naïve gut microbiota on colitis, fecal microbiota transplantation (FMT) confirmed that gut microbiota from GC-K-dosed mice improved intestinal barrier function and allayed experimental colitis. Collectively, our study demonstrated the anti-inflammatory effects of GC-K in a DSS-induced colitis model mainly via regulating gut microbiota.

## Materials and methods

### Materials

Fluorescein isothiocyanate (FITC)-labeled dextran was obtained from Sigma-Aldrich (St. Louis, MO, USA). Antibodies of ZO-1 and Occludin were purchased from Proteintech Group, Inc. (Wuhan, Hubei, China). GC-K was provided by Chengdu Push Bio-technology Co., Ltd. (Sichuan, China).

### Animals and experimental design

Dextran sulfate sodium (DSS; molecular weight, 36–50 kDa) and Sulfasalazine (SASP) were purchased from MP Biomedicals (San Jose, CA, USA) and Aladdin (Shanghai, China), respectively. Female C57BL/6 mice (6 weeks; 17–20 g) were obtained from Hunan SJA Laboratory Animal Co., Ltd. (Hunan, China). The Animal Ethics Committee of Central South University (No. 2020sydw1037) permitted the animal protocol. Mice after acclimation were divided into groups as control, DSS, DSS + LCK (20 mg/kg/day, low dose), DSS + HCK (60 mg/kg/day, high dose), and SASP (200 mg/kg/day, positive control) (Additional file 1: Fig. S1). Mice were fed with 2% DSS in drinking water for 5–7 days, replaced with autoclaved water for another 10–14 days for 3 cycles, and sacrificed on day 42.

### Disease activity index (DAI) and histologic assessment

The DAI was scored every 2 days to evaluate the severity of DSS-induced colitis as described previously [15]. The distal colorectal segments were immersed in 4% paraformaldehyde and stained with hematoxylin and eosin (H&E) to perform a histopathology assay.

### Quantitative real-time PCR analysis

According to previous studies [16], the total RNA of colon tissue was extracted by Trizol reagent. The concentration and purity of total RNA were determined by Nanodrop. SYBR Green Premix Ex TaqII was utilized for qPCR amplification. The relative regulation in target genes after normalization to *β*-action between two groups was calculated using the 2^−ΔΔCt^ method. The qPCR primers of IL-17a, TNF-α, IL-β, IL-10, IL-6, Foxp3, ZO-1, Occludin, E-cadherin, and Mucin-2 were synthesized by Sanggon Biotech (Shanghai, China) (Additional file 2: Table S1).

### Flow cytometry analysis

Spleens were collected, ground, and filtered using 70 μm cell strainers to obtain single-cell suspension. For Th17 cells measurement, cells were counted and stimulated by Leukocyte Activation Cocktail for 4 h. Then, Fixable Viability Dye eFluor ™ 780 (eBioscience) was utilized to identify the Viability of cells. After lysis of the red blood cells, cells were subsequently stained with antibodies as follows, (a) Anti-mouse CD45 Briliant Violet510, Anti-mouse CD3 FITC, Anti-mouse CD4 Briliant Violet421, (b) Anti-mouse IL-17A PE. For Treg cells measurement, cells were accordantly stained with antibodies as follows, (a) Anti-mouse CD45 Briliant Violet510, Anti-mouse CD3 FITC, Anti-mouse CD4 Briliant Violet421, Anti-mouse CD25 PE/Cy™, (b) Anti-mouse Foxp3 APC. All the antibodies were purchased from BD Biosciences (San Jose, CA, USA).

### Western blotting

Proteins were extracted with radioimmunoprecipitation assay (RIPA) lysis buffer and then centrifuged at 12 000 rpm and 4 °C for 15 min. 200 μL of supernatant were subsequently added and mixed well with 40 μL of 5 × SDS-PAGE (100 °C, 10 min). Protein quantification was processed according to the BCA kit procedure. The polyvinylidene difluoride membranes were incubated with the specific primary antibodies, and the appropriate secondary antibodies for 1.5 h at room temperature. The blot was visually analyzed with enhanced chemiluminescence (ECL) substrate and a Bio-Rad imaging system.

### Measurement of FITC-Dextran leakage

Mice were fasted overnight and gavaged with FITC-Dextan (Sigma) at 500 mg/kg. After administration of FITC-Dextran for 4 h, serum was collected to measure the fluorescence intensity.

### 16S ribosomal RNA gene sequencing and data analysis

After sequencing, data processing and quality control were operated using fastq-join (Version: 1.3.1) and pear (v0.9.11) to generate Raw Tags. Cutadapt (version 1.18) was used to get the Clean Tags from the original sequences. Usearch (Version11.0.667) software was used to operate OTU (Operational Taxonomic Units) clustering. Silva (Release132) database was used to pair OTUs sequences for taxonomic analysis. The α-diversity and *β*-diversity of the samples were analyzed by Mothur software and R language tools. LEfSe analysis was performed on LEfSe software to assess the adequate size (LED > 2 or 4) of each distinct taxa or OTU. Correlation analysis was conducted on Wekemo Bioincloud (https://www.bioincloud.tech/).

### Bacterial strains and growth curve

All the bacterial strains were stored in 30% glycerol at − 80 °C until use. The bacteria were divided into the administration group (500 mg/mL or 20 μmol/mL of GC-K) and the control group (DMSO). Their growth dynamics were detected in an anaerobic chamber with mixed anaerobic gas (5% carbon dioxide, 5% hydrogen, 90% nitrogen).

### Fecal microbiota transplantation

FMT was performed according to an established protocol with an appropriate modification [17]. Stools from mice treated with HCK or DSS were collected and stored at -80 °C. 100 mg of stools from donor mice were re-suspended in 1 mL of sterile saline. The solution was centrifuged at 600×*g* for 5 min to obtain the supernatant, which was then centrifuged at 12 000×*g* for 10 min. The pellet was re-suspended and used as transplant bacteria. Mice were fed with antibiotic water (0.5 g/L of ampicillin, 0.5 g/L of metronidazole, 0.5 g/L of neomycin, and 0.25 g/L of vancomycin) for 5 days. Transplantation was performed by oral gavage of 200 μL of bacterial fluid 3 times a week.

### Statistical analysis

The data were expressed as the mean ± SEM and analyzed by GraphPad Prism 8.0 and SPSS software (Version 23). Significant differences between the two groups were evaluated by the two-tailed unpaired Student’s *t*-test, one-way ANOVA, or Kruskal–Wallis with the non-normal or non-parametric distribution. The statistical difference between the model and control group was denoted by "#", as well as the treatments and model groups by "*". The level of significance was set at *p* < 0.05 (* *p* < 0.05, ** *p* < 0.01, *** *p* < 0.001, and **** *p* < 0.0001, the same as "#").

## Results

### GC-K alleviated DSS-induced chronic colitis in mice

**Fig. 1:**
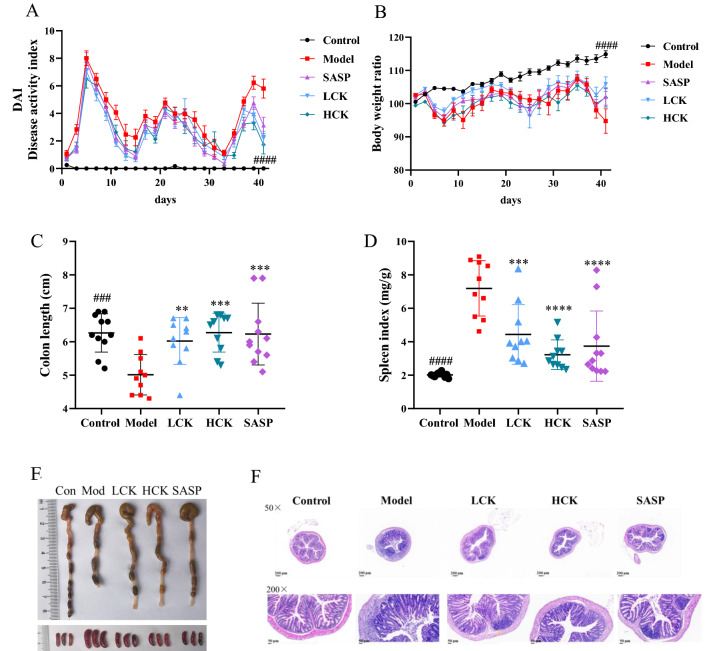
GC-K alleviated DSS-induced chronic colitis. **A** Disease Activity Index during mouse model development; **B** Relative bodyweights during mouse model development; **C** Colon length in each group; **D** Spleen weight in each group (n = 10, 11). **E** Representative colon and spleen pictures from each group. **F** Representative H&E staining of colon tissue sections from each group. (** *p* < 0.01, *** *p* < 0.001 and **** *p* < 0.0001 vs. model group; ^###^ *p* < 0.001 and ^####^ *p* < 0.0001 vs. model group.)

Chronic DSS treatment caused a reduction in body weight and an increase in DAI, which was reversed after being treated with GC-K (Fig. 1A). The body weight showed a slight increase with GC-K treatment (Fig. 1B). GC-K improved colon shortening, reduced spleen weight, and exhibited less immune cell infiltration and tissue damage by comparing with DSS Group (Fig. 1C–F).

**Fig. 2:**
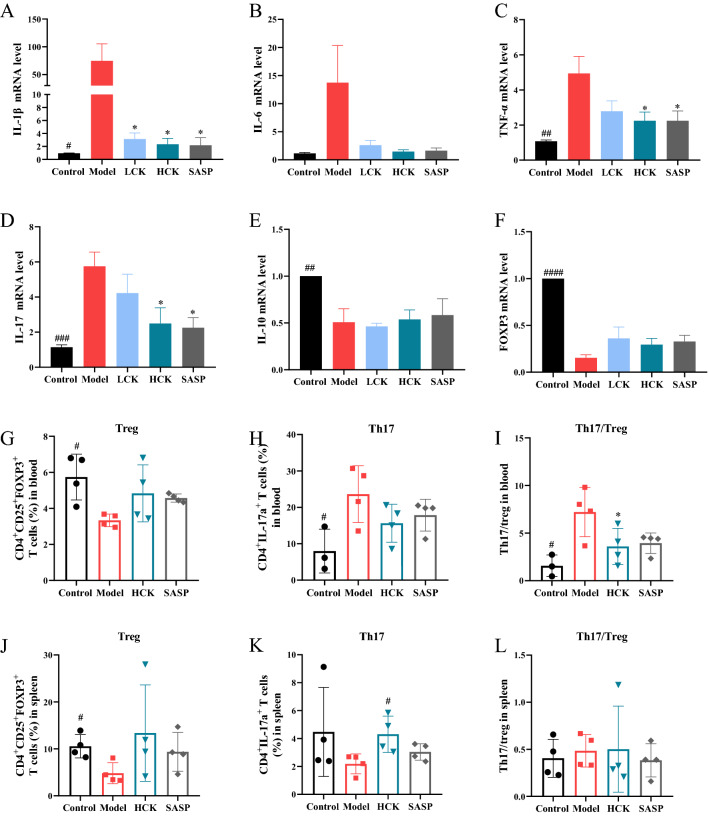
GC-K decreased inflammatory cytokines production during DSS-induced colitis. Inflammatory factors IL-1β (**A**), IL-6 (**B**), TNF-α (**C**), IL-17a (**D**), IL-10 (**E**) and transcription factor Foxp3 (**F**) in mouse colon measured by RT-PCR (n = 4, 5). Flow cytometry analysis of Treg cells (CD3^+^CD4^+^CD25^+^Foxp3^+^) in blood (**G**) and spleen (**J**). Flow cytometry analysis of Th17 cells (CD3^+^CD4^+^IL-17a^+^) in blood (**H**) and spleen (**K**). Flow cytometry analysis of Th17/Treg cells in blood (**I**) and spleen (**L**). (* *p* < 0.1 vs. model group; ^#^ *p* < 0.1, ^##^ *p* < 0.01, ^###^ *p* < 0.001 and ^###^ *p* < 0.0001 vs. model group.)

DSS-induced upregulations of pro-inflammatory cytokines such as IL-1β, IL-17a, TNF-α, and IL-6 were significantly downregulated by GC-K (Fig. 2A–D). In addition, the expression of anti-inflammatory cytokine IL-10 was reduced considerably by DSS (Fig. 2E), but neither GC-K nor SASP had any effect on the production of IL-10. GC-K down-regulated the expression of Foxp3 increased by DSS without significance (Fig. 2F). Th17 cells in the blood were significantly increased after modeling, while GC-K effectively reduced the quantities of Th17 cells and restored the Th17/Treg ratio (Fig. 2G–L). Meanwhile, the amounts of Treg cells in the blood and spleen were recovered with GC-K treatment.

**Fig. 3:**
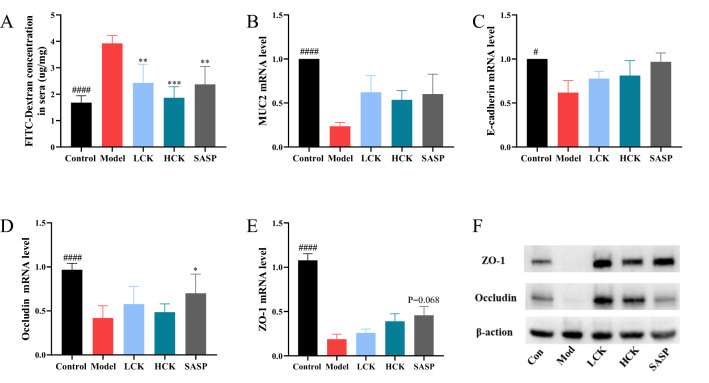
GC-K improves intestinal barrier during DSS-induced colitis. Intestinal leakage measured by FITC-Dextran concentration in serum (**A**); Mucin MUC2 (**B**) and intestinal tight junction proteins E-cadherin (**C**), Occludin (**D**) and ZO-1 (**E**) in each group measured by RT-PCR (n = 4, 5). ZO-1 and Occludin in colon of different groups measured by Western blotting (**F**). (* *p* < 0.1, ** *p* < 0.01 and *** *p* < 0.001 vs. model group; ^#^ *p* < 0.1 and ^####^ *p* < 0.0001 vs. model group.)

The concentration of FITC-dextran was increased in model group, while SASP and GC-K significantly reduced the concentration of dextrose in the plasma (Fig. 3A). Furthermore, GC-K reversed the expressions of Occludin, ZO-1, E-cadherin and MUC2, which implied that mucus layer thickness was restored (Fig. 3B–F).

### Alterations of the gut microbiome by GC-K treatment

**Fig. 4:**
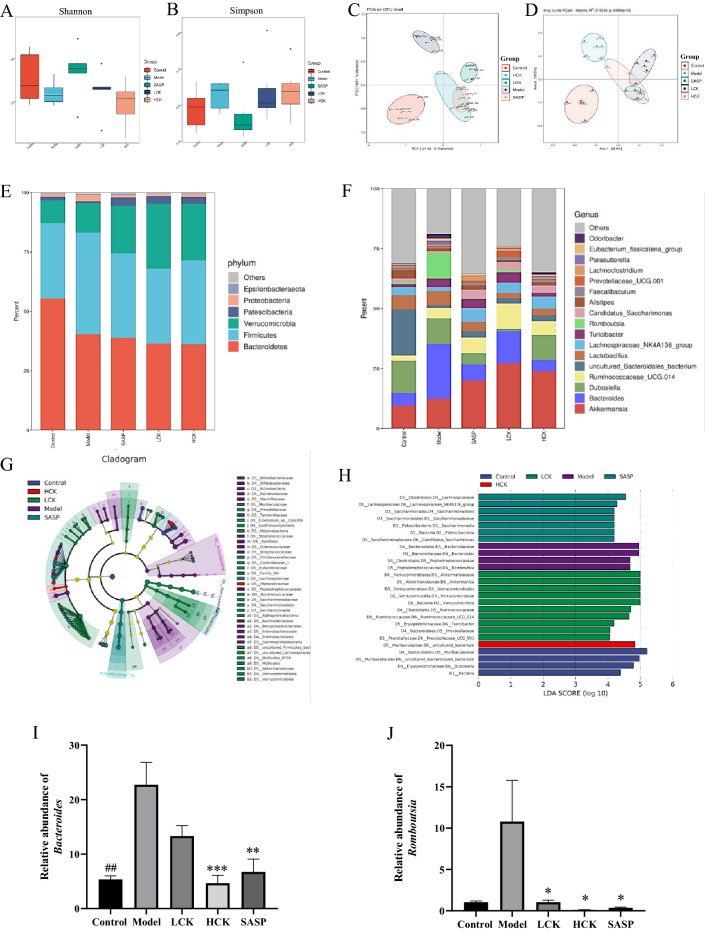
16S rDNA sequencing revealed altered microbiota composition after GC-K treatment (n = 6). The α-diversity of each group measured by Shannon (**A**) and Simpson (**B**) index. PCA score plot analysis based on the relative abundance of OTUs (**C**) and bray curtis-based PCoA analysis (**D**) were used to evaluate the β-diversity of each group. Relative abundance of microbial taxa was determined at the phylum (**E**) and genus (**F**) level. Taxonomic cladogram obtained (**G**) and linear discriminant analysis score (**H**) from linear discriminant analysis effect size analysis of 16S rRNA sequences. Relative abundance of *Bacteroides* (**I**) and *Rombsia* (**J**) in each group. (* *p* < 0.1, ** *p* < 0.01 and *** *p* < 0.001 vs. model group; ^##^ *p* < 0.1 vs. model group.)

The α-diversity showed no significant difference among all groups (Fig. 4A, B). The *β*-diversity revealed distinct clustering of gut bacteria for each group (Fig. 4C, D). The results indicated that the profiles of gut microbiota were significantly different among each group.

Taxonomic histograms of gut bacteria on phylum and genus levels were shown in Fig. 4E, F. The phylum analysis revealed that GC-K increased the relative abundances of Verrucomicrobia and Patescibacteria and decreased Proteobacteria in DSS-induced colitis mice (Fig. 4F and Additional file 1: Fig. S3). In contrast, GC-K significantly increased the relative lots of *Akkermansia*, *Candidatus_Saccharimonas*, and *Ruminococcaceae_UCG-014*, and decreased *Bacteroides* and *Rumboutsia* in genus levels (Additional file 1: Fig. S4). The biomarkers for the Model group cluster were *Bacteroides* and *Rumboutsia*, while the biomarkers of the LCK group cluster were *Akkermansia*, *Ruminococcaceae_UCG-014* and *Turicibacter* (Fig. 4G and H). The results showed that GC-K ameliorated gut microbiota dysbiosis in DSS-induced mice by significantly decreasing the abundance of *Bacteroides*.

To examine whether GC-K directly suppressed the growth of *Bacteroides *in vitro, the growth curves of 7 strains of *Bacteroides* co-incubated with GC-K were monitored (Additional file 1: Fig. S5). GC-K directly suppressed the growth of *B. vulgatus *in vitro, but had no significant effects on other strains of *Bacteroides*.

### The transplant of GC-K-induced microbiota relieved colitis

**Fig. 5:**
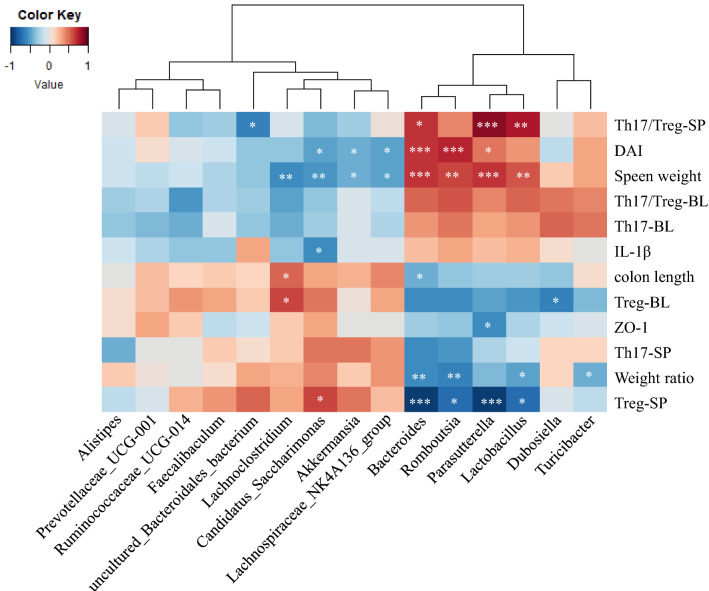
Spearman correlation analysis between top 15 Taxonomic of gut microbiota and evaluation indexes of inflammatory bowel disease. (* *p* < 0.1, ** *p* < 0.01 and *** *p* < 0.001 vs. model group.)

As shown in Fig. 5, a correlation map was constructed to discriminate the specific bacteria correlated with colitis disease indicators. Higher correlation scores indicated that the GC-K-driven gut bacteria potentially reversed colitis disease indicators.

**Fig. 6:**
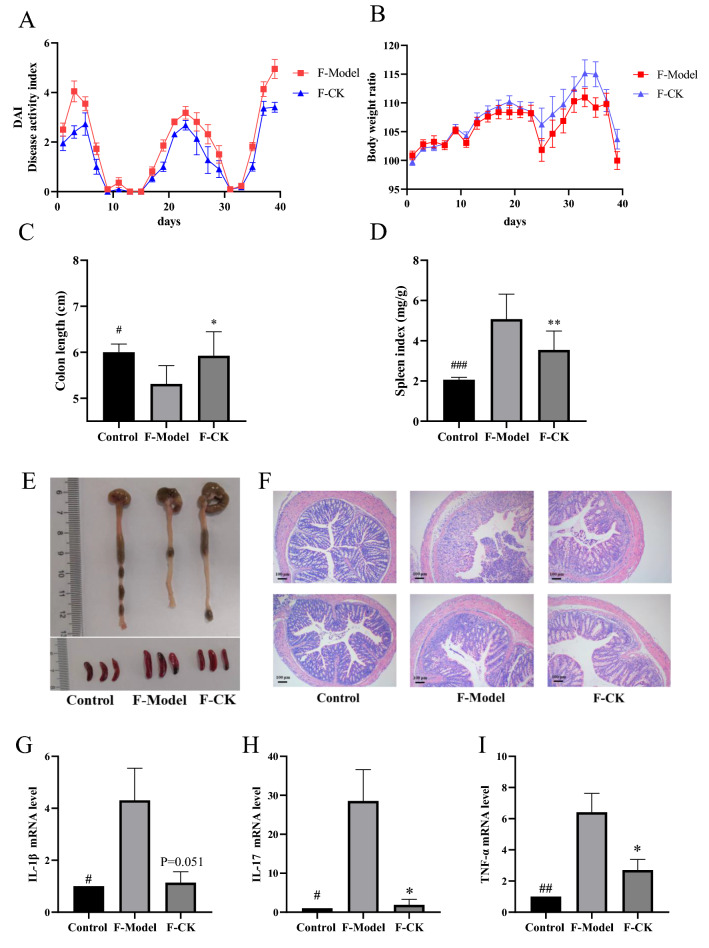
FMT alleviated DSS-induced chronic colitis. **A** Disease Activity Index during mouse model development. **B** Relative bodyweights during mouse model development. **C** Colon length in each group. **D** Spleen weight in each group (n = 6–9). **E** Representative colon and spleen pictures from each group. **F** Representative H&E staining of colon tissue sections from each group. Inflammatory factors IL-1β (**G**), TNF-α (**H**) and IL-17a (**I**) in mouse colon measured by RT-PCR, n=4. (* *p* < 0.1 and ** *p* < 0.01 vs. model group; ^#^ *p* < 0.1, ^##^ *p* < 0.01 ^###^ *p* < 0.001 vs. model group.)

**Fig. 7:**
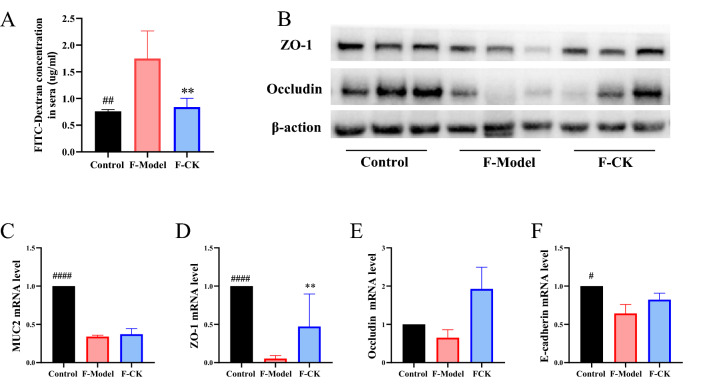
FMT improves intestinal barrier during DSS-induced colitis. Intestinal leakage measured by FITC-Dextran concentration in serum (**A**) (n = 4). ZO-1 and Occludin in colon of different groups measured by Western blotting (**B**) Mucin MUC2 (C) and intestinal tight junction proteins ZO-1 (**D**), Occludin (**F**) and E-cadherin (**G**) in each group measured by RT-PCR (n = 3). (* *p* < 0.1, ** *p* < 0.01, *** *p* < 0.001 and **** *p* < 0.0001 vs. model group; ^##^ *p* < 0.01 vs. model group.)

FMT significantly alleviated DSS-induced colitis and decreased immune cell infiltration in colon tissue, and inflammatory factors expression of IL-1β, TNF-α and IL-17a (Fig. 6). Similar to the donor mice, the quantities of Treg and Th17 cells in the spleen were decreased significantly with DSS administration and increased after treated with GC-K (Additional file 1: Fig. S7). Furthermore, the FITC-dextran leakage experiment confirmed that FMT protected gut leakage. In addition, the expressions of Occludin, ZO-1, MUC2, and E-cadherin were increased, which indicated intestinal barrier function was restored (Fig. 7).

## Discussion

Gut microbiota plays a vital role in maintaining body health [18]. Numerous studies have demonstrated that the gut microbiota is correlated to the occurrence and development of IBD [19], and the abundance and diversity of the gut microbiota of UC patients are decreased [20, 21]. Therefore, the regulation of gut microbiota has become a new therapeutic strategy of colitis.

GC-K is the primary metabolite of the protopanaxadiol ginsenosides bio-converted by gut microbiota [10, 11, 22], which plays a beneficial role in colitis [12]. Recently, natural products have been discovered to interplay with gut microbiota in vivo [23, 24]. Moreover, our previous work has confirmed GC-K suppressed the tumor growth of AOM/DSS-induced colitis-associated CRC via regulating gut microbiota [9]. Thus, the purpose of this work was to determine the role of gut microbiota in the treatment of UC with GC-K.

GC-K relieved the symptoms of weight loss and colon length shortening in DSS-induced mice. Compared with SASP, GC-K has advantages in weight and DAI scores. In colonic histopathology, GC-K was observed to reduce histologic inflammation. In addition, GC-K significantly decreased the mRNA expression of inflammatory cytokines (TNF-α, IL-6, IL-1β, and IL-17a) in colon tissue. The intestinal mucosal barrier could isolate the intestinal lumen from the environment to prevent the invasion of bacteria and toxic substances [25]. Still, an increase in proinflammatory cytokines further damages intestinal mucosal barrier function to make colitis worse [26, 27]. Occludin, ZO-1, and E-cadherin are crucial for connecting individual epithelial cells and maintaining the integrity of the epithelium [25]. The intestinal mucus layer is a protective gel-like substance covering the surface of the intestinal mucosa, which is the first barrier in the intestinal lumen [28]. Our experiment showed that DSS-induced colitis decreased the mRNA expressions of Occludin, ZO-1, E-cadherin, and MUC2 in colon tissue, which could be reversed by GC-K treatment.

According to 16S rRNA sequencing analysis, lower abundances of *Lachnospiraceae_NK4A136_group* and *Candidatus_Saccharimonas* and higher abundances of *Bacteroides*, *Romboutsia*, and *Turicibacter* were observed in the DSS group than in the control group, which were consistent with previous reports [29, 30]. GC-K reversed the intestinal dysbacteriosis caused by DSS. In addition, the abundance of *Bacteroides* and *Romboutsia* in GC-K significantly decreased. Spearman correlation analysis showed that *Bacteroides* mainly was correlated with the disease indicators of UC. In vitro, co-incubation experiments showed that GC-K significantly inhibited the growth of *B. vulgatus*. Studies have shown that the abundance of *B. vulgatus* in UC patients is increased substantially, and the proteases from *B. vulgatus* could be involved in UC pathogenesis [8]. The above results indicate that GC-K cotrolled the structure and abundance of gut bacteria and reduced the quantity of *Bacteroides*. However, the detailed mechanisms of GC-K regulated *Bacteroides spp*. remain unclear and need further investigation.

Finally, to further explore whether GC-K relieved colitis by modulating gut microbiota, we used FMT to verify the anti-colitis of GC-K in the DSS model. Before FMT, the pseudo-sterile mice were constructed with antibiotics, since gut microbiota may lead to colonization resistance. FMT relieved colonic inflammation and decreased intestinal permeability, which reproduced the anti-colitis effects of GC-K. Collectively, our data supported GC-K ameliorated colitis via regulating gut microbiota.

## Conclusion

Our study evidenced that the anti-colitis effect of GC-K was related to gut microbiota, which was modulated by GC-K. Fecal microbiota transplantation experiments demonstrated that GC-K-driven gut microbiota significantly relieved DSS-induced colitis. In conclusion, GC-K showed anti-colitis effects via regulating gut microbiota, but the suitable mechanism needs further study.

## Supplementary Information

**Additional file 1: Fig. S1.** The animal experimental protocol. DSS, dextran sulfate sodium; GC-K, ginsenoside compound K. **Fig. S2.** The frequency of Foxp3 + Treg cells among the CD4 + T cells (CD3 + CD4 + CD25 + cells) in spleen(A) and blood(B) of mice detected by flow cytometry; The frequency of IL-17a + Th17 cells among the CD4 + T cells (CD3 + CD4 + cells) in spleen(C) and blood(D) of mice detected by flow cytometry (n = 4, 5). **Fig. S3.** The relative abundance of the top 5 species at the phylum level in each group. Relative abundance of Bacteroidetes(a), Firmicutes(b), Verrucomicrobia(c), Patescibacteria(d) and Proteobacteria(e) in each group (n = 6). (* *p* < 0.1 and ** *p* < 0.01 vs. model group.) **Fig. S4.** Relative abundance of *Akkermansia*(a), *Dubosiella*(b), *Lachnospiraceae_NK4A136*(c), *Ruminococcaceae_UCG-014*(d), *Turicibacter*(e) and *Candidatus_Saccharimonas*(f) in each group (n = 6). (* *p* < 0.1 and ** *p* < 0.01 vs. model group.) **Fig. S5** Effects of GC-K on the growth of representative strains in *Bacteroides*. (*** *p* < 0.001 vs. model group.) **Fig. S6.** Fecal microbial DNA concentration of mice before and after antibiotic administration. (** *p* < 0.01 vs. model group.) **Fig. S7.** Flow cytometry analysis of Treg cells (CD3 + CD4 + CD25 + Foxp3 +) and Th17 cells (CD3 + CD4 + IL-17a +) in spleen of mice in FMT group (n = 4). (* *p* < 0.1 and **** *p* < 0.0001 vs. model group; ^###^ *p* < 0.001 vs. model group.)

**Additional file 2: Table S1.** The primer sequences used in real-time qPCR assays in colonic tissue.

## Data Availability

The research data generated from this study are included within the article and additional files.

## Abbreviations

16S rRNA: 16S ribosomal RNA
GC-K: Ginsenoside compound K
LDA: Linear discriminative analysis
LEfSe: The linear discriminative analysis effect size
PCoA: Principal component analysis
PC: Principal component
RT-PCR: Real-time polymerase chain reaction
QIIME: Quantitative Insights Into Microbial Ecology
