# Supplementary material for: Effect of ginsenoside compound K on alleviating colitis via modulating gut microbiota
Source: Chin Med. 2022 Dec 28;17:146. doi: 10.1186/s13020-022-00701-9 (PMC9795722; doi:10.1186/s13020-022-00701-9)
Supplement: Supplementary file 1 — Additional file 1: Fig. S1. The animal experimental protocol. DSS, dextran sulfate sodium; GC-K, ginsenoside compound K. Fig. S2. The frequency of Foxp3 + Treg cells among the CD4 + T cells (CD3 + CD4 + CD25 + cells) in spleen(A) and blood(B) of mice detected by flow cytometry; The frequency of IL-17a + Th17 cells among the CD4 + T cells (CD3 + CD4 + cells) in spleen(C) and blood(D) of mice detected by flow cytometry (n = 4, 5). Fig. S3. The relative abundance of the top 5 species at the phylum level in each group. Relative abundance of Bacteroidetes(a), Firmicutes(b), Verrucomicrobia(c), Patescibacteria(d) and Proteobacteria(e) in each group (n = 6). (* p < 0.1 and ** p < 0.01 vs. model group.) Fig. S4. Relative abundance of Akkermansia(a), Dubosiella(b), Lachnospiraceae_NK4A136(c), Ruminococcaceae_UCG-014(d), Turicibacter(e) and Candidatus_Saccharimonas(f) in each group (n = 6). (* p < 0.1 and ** p < 0.01 vs. model group.) Fig. S5 Effects of GC-K on the growth of representative strains in Bacteroides. (*** p < 0.001 vs. model group.) Fig. S6. Fecal microbial DNA concentration of mice before and after antibiotic administration. (** p < 0.01 vs. model group.) Fig. S7. Flow cytometry analysis of Treg cells (CD3 + CD4 + CD25 + Foxp3 +) and Th17 cells (CD3 + CD4 + IL-17a +) in spleen of mice in FMT group (n = 4). (* p < 0.1 and **** p < 0.0001 vs. model group; ### p < 0.001 vs. model group.) [file 13020_2022_701_MOESM1_ESM.pptx]

## Slide 1
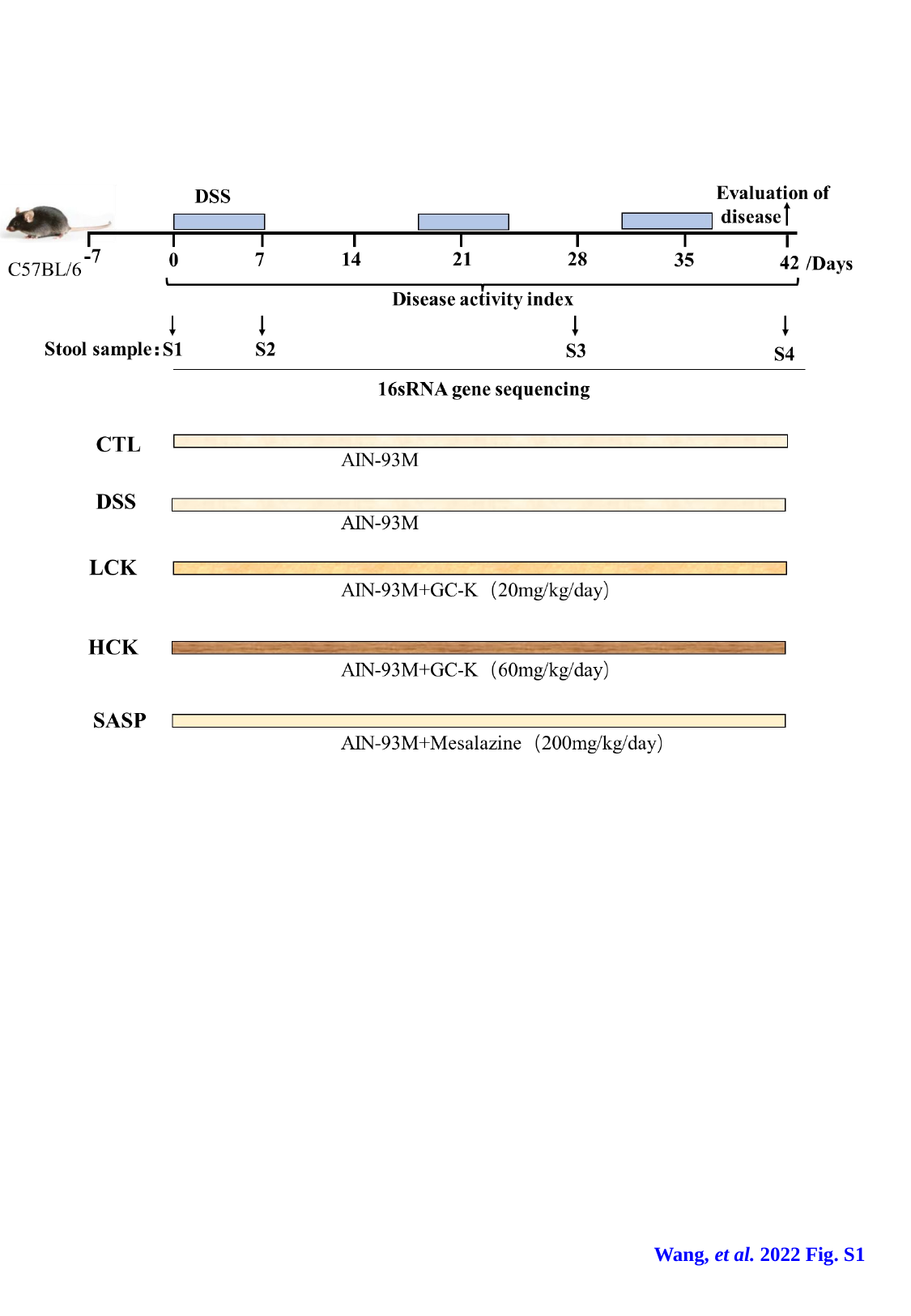

Wang, et al. 2022 Fig. S1

## Slide 2
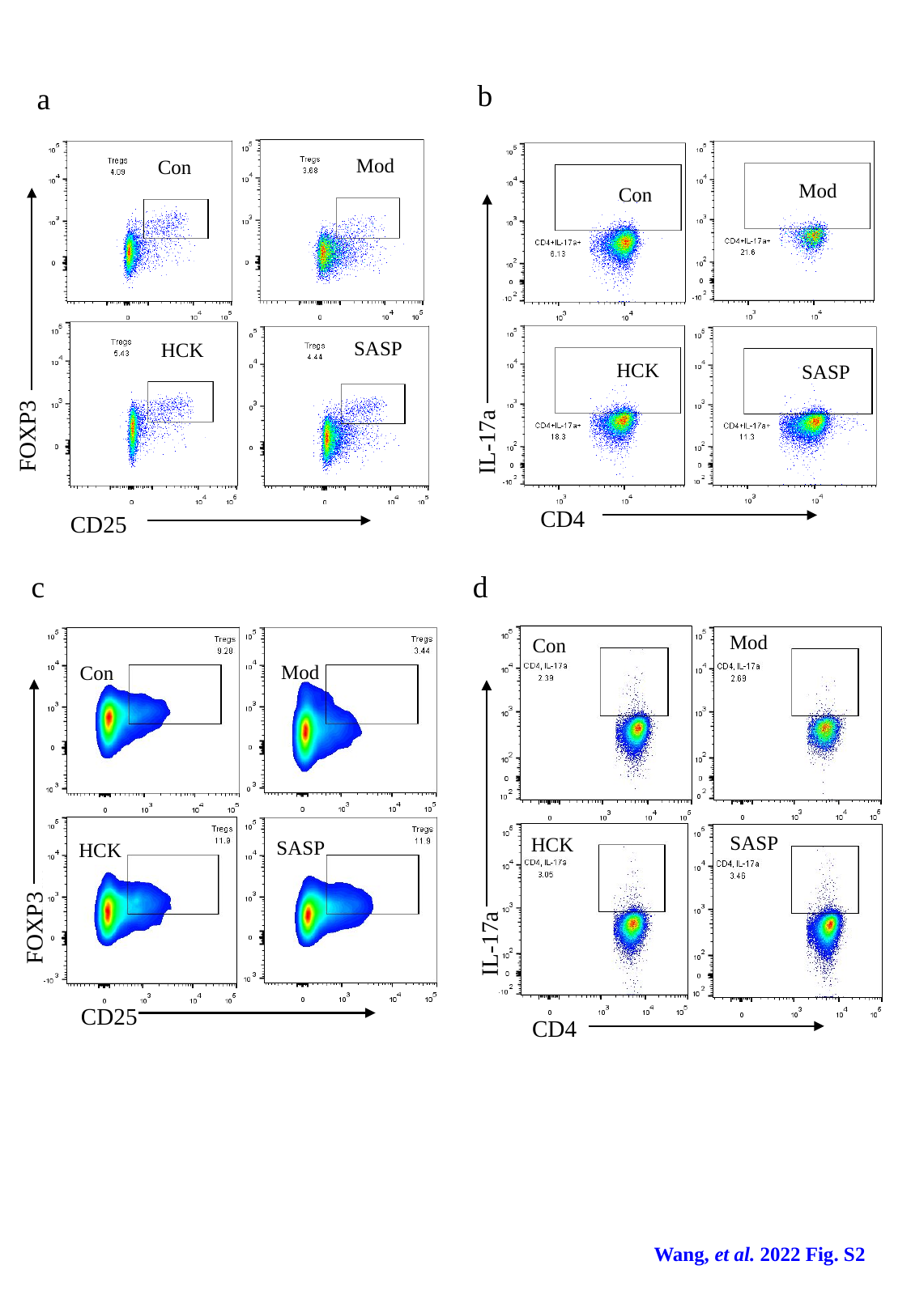

b
a
Mod
Con
SASP
HCK
FOXP3
CD25
Mod
Con
HCK
SASP
IL-17a
CD4
d
c
Mod
Con
SASP
HCK
IL-17a
CD4
Mod
Con
SASP
HCK
FOXP3
CD25
Wang, et al. 2022 Fig. S2

## Slide 3
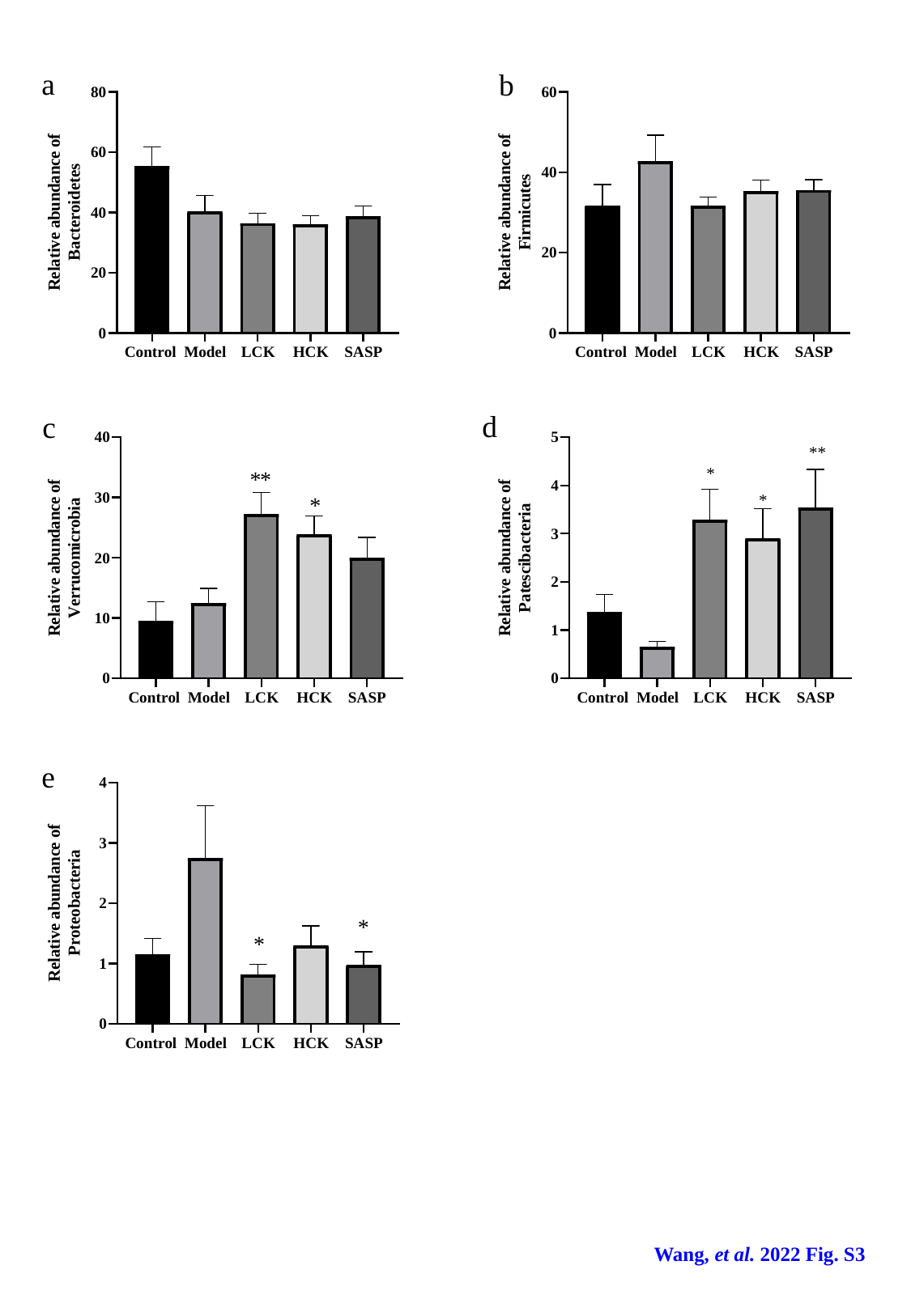

a
b
d
c
e
Wang, et al. 2022 Fig. S3

## Slide 4
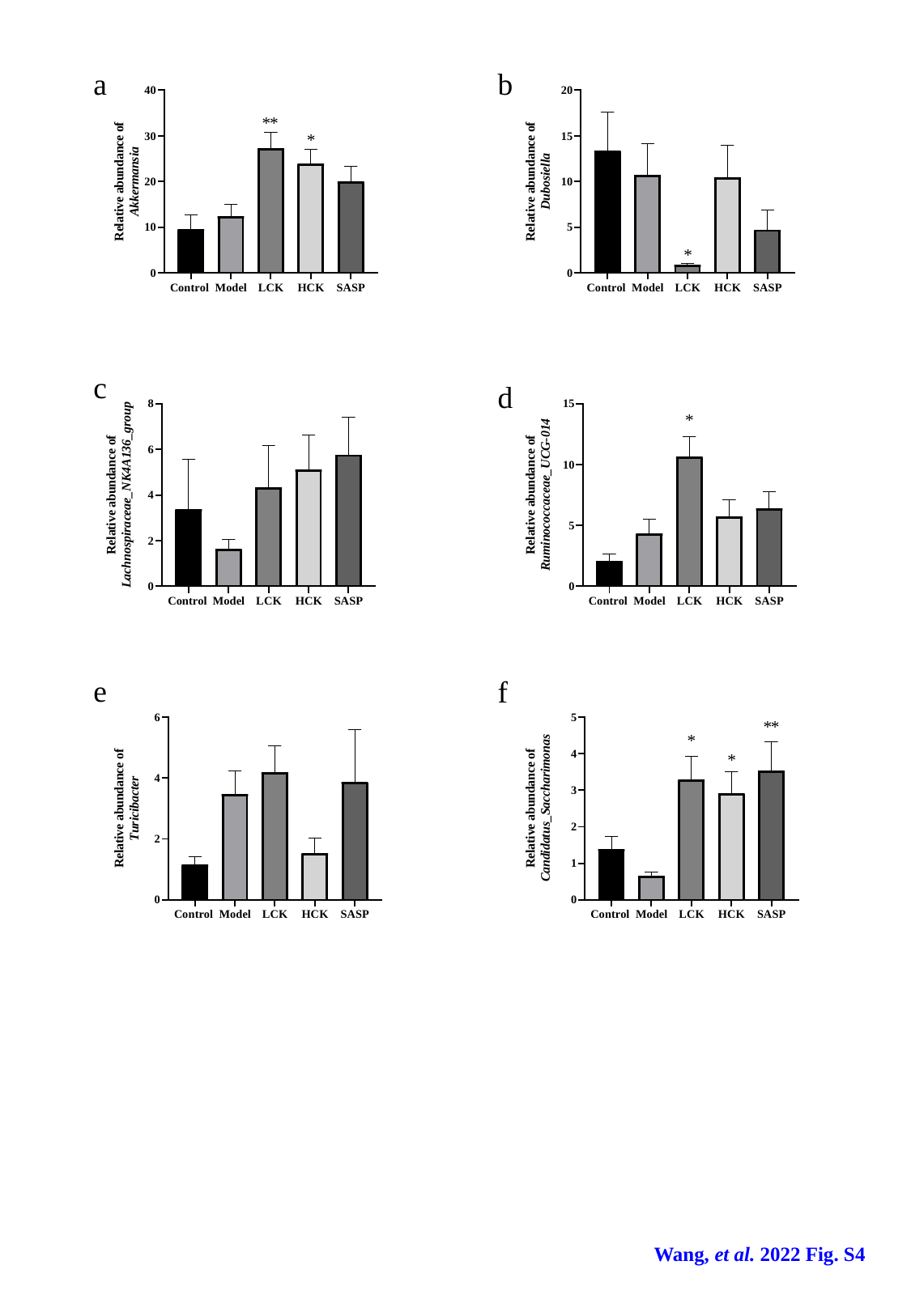

b
a
c
d
e
f
Wang, et al. 2022 Fig. S4

## Slide 5
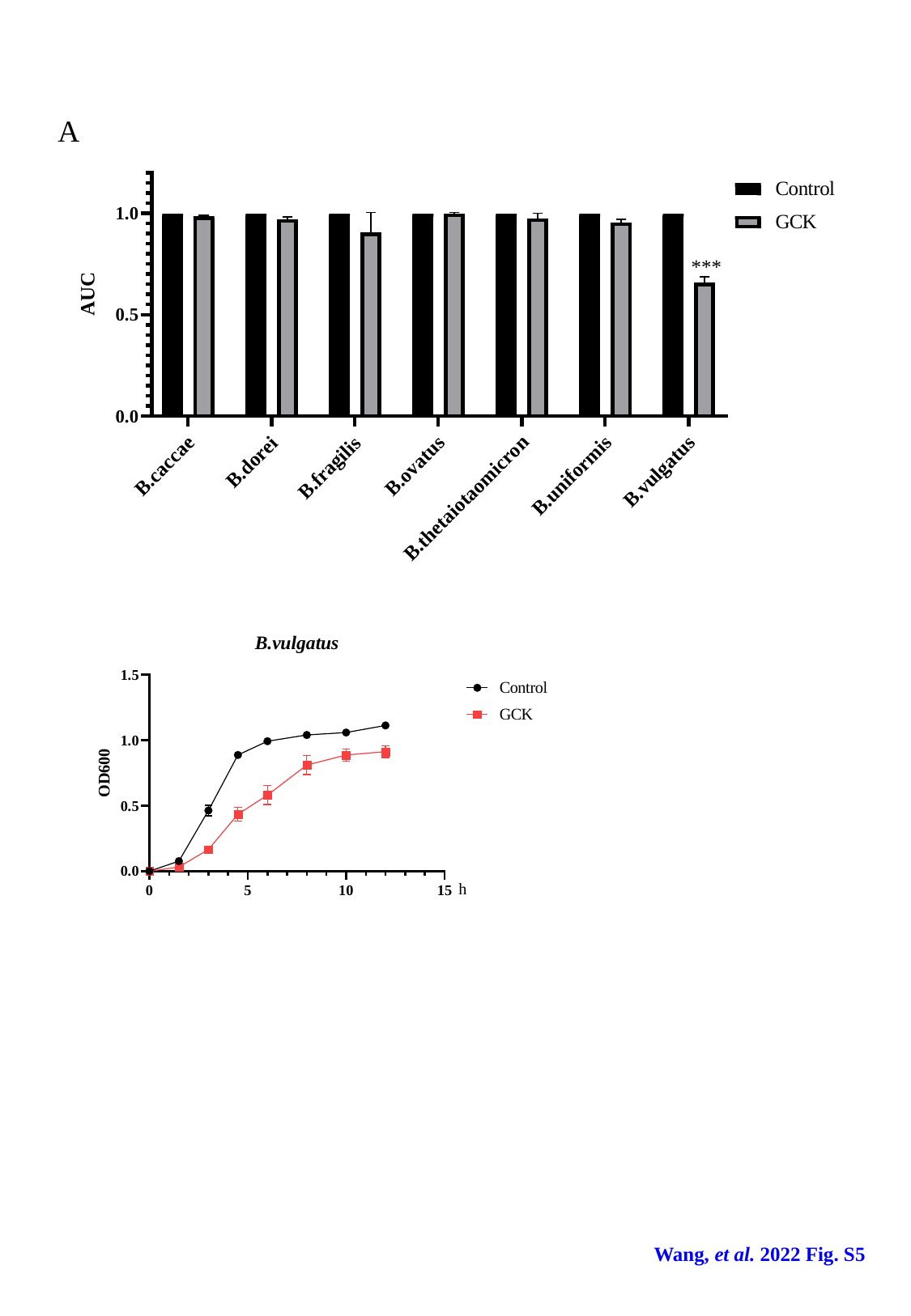

A
Wang, et al. 2022 Fig. S5

## Slide 6
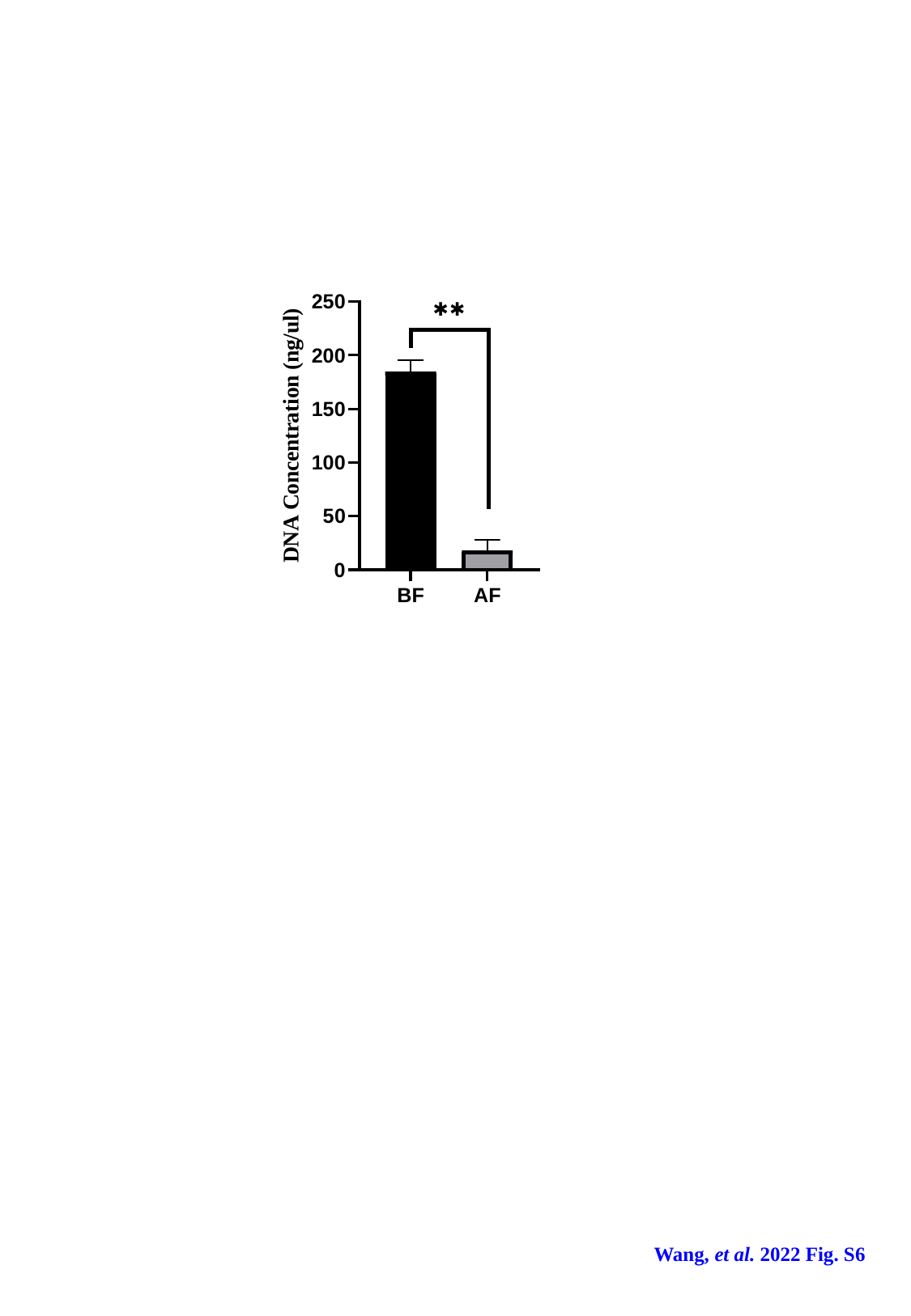

Wang, et al. 2022 Fig. S6

## Slide 7
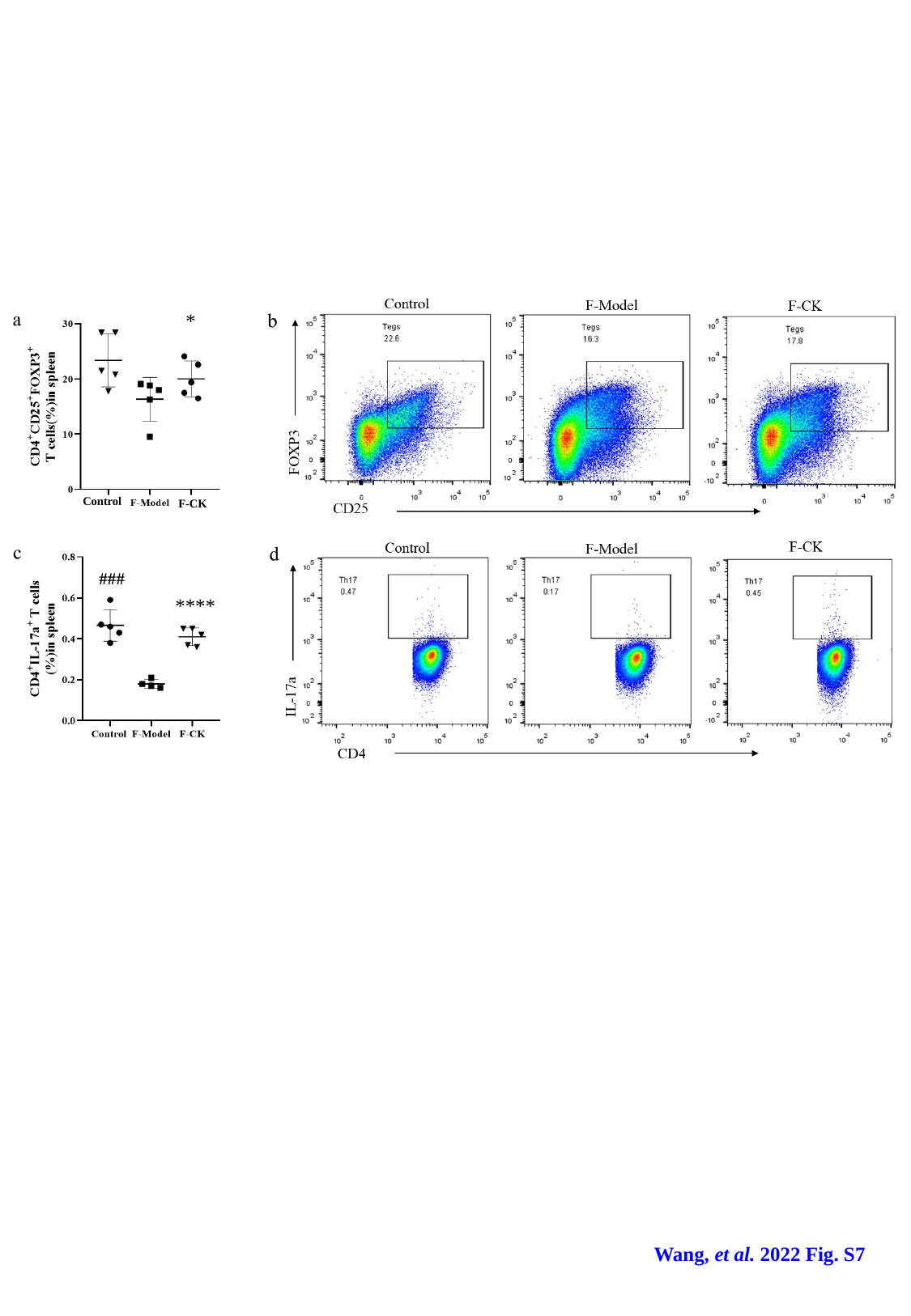

*
Control
F-CK
###
****
Wang, et al. 2022 Fig. S7
