# Supplementary material for: Effect of ginsenoside compound K on alleviating colitis via modulating gut microbiota
Source: Chin Med. 2022 Dec 28;17:146. doi: 10.1186/s13020-022-00701-9 (PMC9795722; doi:10.1186/s13020-022-00701-9)
Supplement: Supplementary file 2 — Additional file 2: Table S1. The primer sequences used in real-time qPCR assays in colonic tissue. [file 13020_2022_701_MOESM2_ESM.docx]

Table S1 The primer sequences used in real-time qPCR assays in colonic tissue

| Gene | Forward Primer | Reverse Primer |
| --- | --- | --- |
| IL-10 | GCTCTTACTGACTGGCATGAG | CGCAGCTCTAGGAGCATGTG |
| IL-17a | TTTAACTCCCTTGGCGCAAAA | CTTTCCCTCCGCATTGACAC |
| IL-1β | GAAATGCCACCTTTTGACAGTG | TGGATGCTCTCATCAGGACAG |
| IL-6 | TAGTCCTTCCTACCCCAATTTCC | TTGGTCCTTAGCCACTCCTTC |
| TNF-α | CAGGCGGTGCCTATGTCTC | CGATCACCCCGAAGTTCAGTAG |
| Foxp3 | CACCTATGCCACCCTTATCCG | CATGCGAGTAAACCAATGGTAGA |
| ZO-1 | AGATGAACGGGCTACGC | GGAGACTGCCATTGCTTG |
| Occludin | CACGCTTGCCTGGGACAGAG | TCTGTATAGCCTCCGTAGCC |
| E-cadherin | CAGGTCTCCTCATGGCTTTGC | CTTCCGAAAAGAAGGCTGTCC |
| Mucin-2 | AGGGCTCGGAACTCCAGAAA | CCAGGGAATCGGTAGACATCG |
| β-actin | GGCTGTATTCCCCTCCATCG | CCAGTTGGTAACAATGCCATGT |
